# False Data Detection for Fog and Internet of Things Networks

**DOI:** 10.3390/s19194235

**Published:** 2019-09-29

**Authors:** Romano Fantacci, Francesca Nizzi, Tommaso Pecorella, Laura Pierucci, Manuel Roveri

**Affiliations:** 1 Department of Information Engineering, University of Florence, 50139 Firenze, Italy; 2 Dipartimento di Elettronica e Informazione, Politecnico di Milano, 20133 Milano, Italy

**Keywords:** Internet of Things, security, dynamic protection, intelligence for embedded and cyber-physical systems, adaptive systems, fault detection and diagnosis, smart sensor networks

## Abstract

The Internet of Things (IoT) context brings new security issues due to billions of smart end-devices both interconnected in wireless networks and connected to the Internet by using different technologies. In this paper, we propose an attack-detection method, named Data Intrusion Detection System (DataIDS), based on real-time data analysis. As end devices are mainly resource constrained, Fog Computing (FC) is introduced to implement the DataIDS. FC increases storage, computation capabilities, and processing capabilities, allowing it to detect promptly an attack with respect to security solutions on the Cloud. This paper also considers an attack tree to model threats and vulnerabilities of Fog/IoT scenarios with heterogeneous devices and suggests countermeasure costs. We verify the performance of the proposed DataIDS, implementing a testbed with several devices that measure different physical quantities and by using standard data-gathering protocols.

## 1. Introduction

The advent of the Internet of Things (IoT) opens up new vulnerabilities for both security and privacy due to the massive number of resource-constrained devices connected to the Internet by using various technologies.

The IoT paradigm is worsening the overall security issues due to the heterogeneity of connected IoT hardware platforms (i.e., different firmware types, revisions, etc.) and to the variety of network technologies for interconnections (e.g., Bluetooth, 802.15.4, NarrowBand IoT (NB-IoT), etc.), all with potential flaws and vulnerability to attacks. An IoT device (*a thing*) can be a light bulb, a thermostat, a smartphone, a personal computer, or potentially everything. IoT devices have to face many threats originating from the Internet and can also become a source of attacks towards the Internet. Many IoT devices might become easy targets to cyber adversaries due to configuration mistakes, e.g., default password unchanged as for the case of My Friend Cayla, a famous toy [1]), or of unpatched vulnerabilities. A fairly recent example of this issue is a Distributed Denial of Service (DDoS) attack that occurred in 2016 against the service DynDNS [2]: the malware Mirai [3] was responsible of the botnet creation composed by “innocent” IoT devices such as IP cameras, printers, and baby monitors. Jackware is a ransomware version designed specifically for IoTs to hit smart devices, and as a consequence, millions of users would be willing to pay the ransom if unable to carry out daily commands at home or in the car.

Due to a massive number of interconnected devices and their low power and limited processing power, IoT networks need to share data with the Cloud for storage and processing, entailing new security requirements.

Fog Computing is a novel paradigm that complements Cloud Computing by moving storage, computation, and application services from the Cloud towards the edge of network. This is really useful for IoT applications, as in this way, data can be kept local to enable novel and more efficient security and privacy methods. Therefore, this paper suggests Fog unit (FU) for supporting a novel Data Intrusion Detection Systems (DataIDS) to detect malicious activities in IoT end-devices.

**Figure 1:**
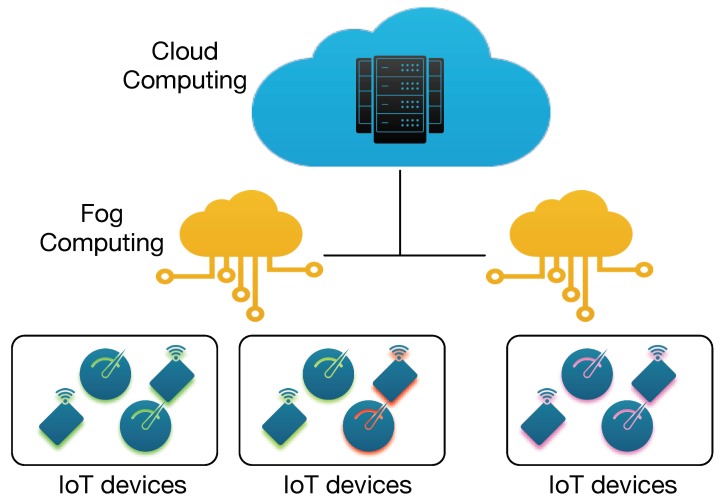
Fog and Internet of Things (IoT) scenarios.

A typical Fog/IoT scenario is shown in Figure 1. IoT devices are organized into clusters, and each cluster is managed by one or more FUs with higher computational power used to locally collect, store, and process data. The FU acts as a bridge between IoT devices and the Cloud, possibly decoupling the IoT-based protocols from the protocols used on the Internet, enabling moreover better energy efficiency. Usually, the devices and network details are masqueraded by an appropriate abstraction level. However, this also implies that the security layer cannot leverage the intrinsic information of the physical system. Performing some security procedures in the Fog enables to leverage the physical system (e.g., the network topology) along with all the information that are usually not transferred to the Cloud. As an example, sensors produce a high number of data readings but only the data subscribers are informed of the readings and usually only when a given threshold is reached. On the other hand, the FU can perform a more accurate and prompt analysis of the IoT system behavior, can react faster than an equivalent Cloud-based solution, can minimize the amount of data that is exchanged on the Internet, and can prevent or promptly react to an attack with respect to a security action performed on the Cloud, enhancing the IoT network security and privacy.

In IoT deployments, the standard security mechanisms, such as cryptography and authentication, are mandatory. Nevertheless, devices are often vulnerable to a broader attack range due to the particular attack surface (e.g., large number of devices, installation in non-monitored environments, resource contains leading to weaker cryptography, etc.). As a consequence, Intrusion Detection Systems (IDS) are needed.

Usually, IDSs analyze network traffic patterns, packet contents, or systems logs, searching for evidence of security violations mainly at the network layer (e.g., for routing attacks). Sadly, a large class of attacks targeting the IoT data cannot be easily detected by traditional IDSs.

In this paper, we propose a novel IDS, named DataIDS, specifically designed for Fog/IoT networks, based on the analysis of physical (sensed) data to better recognize vulnerabilities against the end-devices. The measurements carried out by sensors are sent to the FU, which locally processes the data streams, and if an anomalous behavior is detected, it can raise an alarm and manage appropriate countermeasures, e.g., to isolate the devices under attack, to discard their data, to authenticate a sensor and its data, or to reconfigure the IP addresses.

The DataIDS distinguishing features are i) the ability to detect a malicious (or false) data injection by analyzing the datastreams acquired by the devices and, ii) at the same time, to find the devices which are currently misbehaving. The key idea is to build a *dependency graph* by analyzing the cross correlation among the respective data streams of sensors and to use that information to highlight any anomalies in the system. This allows to react promptly to a threat with the appropriate actions and/or to trigger further analysis mechanisms aimed at verifying the sensor health conditions. It is worth mentioning that DataIDS can be easily integrated into Fog nodes without significantly impacting their performance by enabling a Fog node to control a very large number of IoT devices and to raise an alarm and related countermeasures if one or more devices are under attack.

To complement the DataIDS approach, we propose a novel attack tree with associate risks, costs, and level of potential system damage. According to the detected threats, the attack tree is a valid method to select the appropriate action to be undertaken, which can span from simply discarding the data of attacked sensors to a full network reconfiguration.

We implemented a test bed to validate the proposed DataIDS performance on real datasets acquired with several sensors measuring different physical quantities when different data injection attacks occurr, such as stuck-at, replay, and sensor replacement.

The reminder of this paper is organized as follows. Section 2 provides a literature overview on IDS for IoT and, in particular, the main differences of our method with similar works. Section 3 analyzes the attack tree to model the possible threats and vulnerabilities of the Fog/IoT system and describes the data-injection attack models considered, while Section 4 shows the proposed solution for detecting and isolating attacks. In Section 5, the experimental results are provided for the considered attacks by using a real test bed, highlighting the effectiveness of the proposed method, and the related countermeasure costs for the attacked sensor are shown. Finally, the conclusions are drawn.

## 2. Related Works

A Fog/IoT system is subjected to attacks both from the Internet and from within the wireless sensor network; therefore, firewalls to isolate the sensitive part of the network and IDSs to detect attacks are needed.

An IDS is a software tol [4,5] that collects and analyzes input data coming from a network, in order to find possibles security breaches. Usually, IDSs are classified in two categories:

- *Signature detection system*. The possible intrusions are identified through traffic patterns and/or predetermined attack signatures. The main benefit of this technique is the high detection reliably. On the other hand, the signature of each known attack should be stored with significant storage and computational costs increasing with the number of attacks. Moreover, the attack signatures database must be always up-to-date.
- *Anomaly detection system*. The IDS compares user behaviors with a model. If the behavior differs from the model, an alarm is raised. It can detect unknown attacks (the so-called *zero-day*) but it requires definition of the model of normal system behavior.

Another IDS classification can be based on the type of data monitored by the IDS: a Network-based IDS (NIDS) analyzes network traffic, while a Host-based IDS (HIDS) monitors a computer (its running programs, application logs, etc.) [6]. The two types can be also used jointly in order to provide a comprehensive networked system protection.

Surveys on different IDS types can be found in Reference [4,6,7,8], highlighting that IDSs mainly work by analyzing log files and/or network traffic patterns. Moreover, most of them are not specifically designed for IoT.

**Figure 2:**
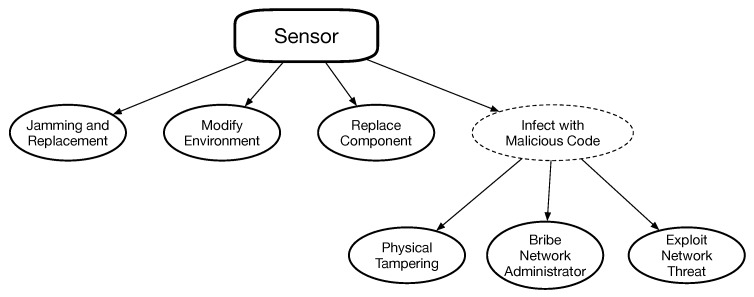
Attack tree for our IoT system.

Applying an NIDS to the IoT scenario raises some noteworthy issues, like the number of traffic flows to be analyzed and the need to collect traffic from multiple network points, which can be extremely costly in a multi-hop network. Moreover, the traffic pattern is not suitable for anomaly-based IDS due to the huge differences in traffic patterns in case of particular events (e.g., a sensor might increase suddenly its sampling rate depending on the environment it is controlling). HDISs are not suitable either due to the limited sensor computational and energy resources. It is possible to add mechanisms to prevent firmware tampering, but it is not a common solution for commercial systems. Furthermore, NDISs and HDISs cannot detect a wide range of attacks highlighted in the attack tree in Figure 2 and, in particular, the attacks targeting (or consequence of) a change in the physical world, i.e., environment modifications (e.g., placing a heat source near a sensor), modifications to the device hardware components, etc.

In the literature, IDSs designed for IoT mainly consider attacks at the network layer (usually routing attacks). Examples are SVELTE [9], used to detect sinkhole and selective-forwarding attacks; Complex Event Processing (CEP) [10], able to analyze the information streams to detect events in real-time; or the approach presented in Reference [11], able to detect Denial of Service Attacks (DoS) targeting the Routing Protocol for Low-Power and Lossy Networks (RPL).

The authors in Reference [12] propose an algorithm based on four phases, i.e., initialization, estimation, similarity check, and characterization. During the first phase, an estimation model is produced and a similarity check is defined. The second phase, that is the core of the overall system, extracts and iteratively aggregates the estimates of the measurements (following the information defined in the first step) that are then sequentially analyzed by two different tests. When a change is detected, the characterization phase is activated to identify the compromised sensor. This solution encompasses only a linear fixed model among acquired measurement and is applied only to homogeneous measurements (hence, gathered by the same type of sensors). Rather, our proposed DataIDS can work on different heterogeneous measurements.

An IDS for the detection of malicious data injection based on wavelet transform is proposed in Reference [13]. Even in this case, the algorithm is dived into three phases: detection, characterization, and diagnosis. In the first phase, an anomaly score based on the wavelet coefficient is sequentially analyzed over time, inspecting for changes by means of a thresholding mechanism. When a change is detected, the next characterization and diagnosis phases are activated. Such solution focuses only on the spatial correlation not exploiting the temporal correlation present in the acquired data as in the detection phase of our proposed DataIDS. In addition, such a solution requires knowledge of the conditions during the “event” target and relies on information about the position of the nodes.

In Reference [14], the authors present an anomaly behavior analysis IDS able to detect attacks in a smart home system. This framework builds sensor profiles by using the Discrete Wavelet Transform method on the sent data, and the euclidean distance (ED) is used for comparison with the reference profiles obtained during the offline training phase to detect abnormal behaviors. As our proposed algorithm, these processes are performed during the run time. The major difference with our DataIDS is the learning phase: DataIDS does not need to know the data nature provided during training because the dependency graph (see Section 4) could have measurements of different types (for example, humidity and temperature). Then, this leads to have a more flexible system in the monitoring phase: the devices must monitor that their behavior is consistent with the other members of the dependency graph (if a sensor evaluates a change and the other ones are the same, it means that it is really the environment changing).

## 3. Attack Tree and Attack Models

A threat model and the associated risk management help to find security policies and countermeasures that could prevent an attack or mitigate its outcomes [15]. As a matter of fact, without a proper threat model, the system security cannot be guaranteed because some threats could be underestimated or, on the other hand, some threats could be overestimated, leading to unnecessary security restrictions and extra costs. A successful risk management process has to also balance the cost of security techniques and the system usability for each potential attack. Therefore, an optimal security system is the one where implementation does not become more expensive than the possible damage of the attack that is being prevented.

### 3.1. Attack Tree

We consider the attack tree to model the possible threats and vulnerabilities of our system. The term attack tree was introduced by Schneier in Reference [16] and represents a tool to evaluate the effectiveness of an attack and appropriateness of a countermeasure, depending on the attack type and extent. An attack tree describes the possible attacks to the network system through a graphical tree structure where the root node is the target of the attacker (the goal) and the leafs are all the possible (and impossible) means to compromise the target (i.e., the attacks) [17]. It is worth noticing that several roots (targets) might exist in the same system. In this case, multiple attack trees must be considered.

Building an attack tree consists of four main steps:

1. Define the main attack goal.
2. Decompose the main attack goal into sub-targets.
3. Assign values to the leafs.
4. Calculate the cost of an attack.

The values assigned to the attack tree leaves can represent different properties of the attack, and they can be boolean or continuous on a specified range. As an example of boolean properties, we can list if the attack is easy, if it is expensive, if particular skills of the attacker are required, etc. Continuous values can represent the attack cost, its likelihood, the time required to perform the attack, etc. Moreover, if more than one condition must be fulfilled to perform an attack, nodes can be connected, e.g., in case of an attack that could be exploited only after a different one has been performed. The resulting values can be used to make assumptions about the attack and the attacker, i.e., to build the threat model.

The attack tree evaluation is helpful in risk management because, if an attack is easy or the cost is low, its occurrence is likely or, if the cost of countermeasures is much higher than the attack outcomes, the attack can be ignored.

The attack tree for our Fog/IoT system is shown in Figure 2. We only highlighted the possible attacks on the IoT domain without considering the well-known vulnerabilities of FUs and gateways.

Looking at Figure 2, we notice that some attacks can be detected by “traditional” systems, such as IDS, logging programs, etc. but that some attacks are specific and do not leave any trace in the parameters analyzed by the techniques mentioned above, as explained in Reference [18]. Therefore, we need a technique to detect possible attacks by analyzing alternative parameters, such as data measurements sent by sensors as in the proposed DataIDS. The advantages of our approach is summarized in Table 1.

The sensors can be classified according to the importance of the sensed value (e.g., if the reading cannot be inferred from other sensors, if the reading is particularly critical for the IoT application, etc.) or the topology of the network (e.g., if the sensor node acts as a router in a multi-hop topology).

The threats have to be analyzed according to their likelihood and damage factors. In the damage and attack costs, we estimate respectively the cost of the countermeasure and the difficulty for an attacker to successfully execute a particular attack.

To evaluate the damage cost, we consider the following factors:

- The node position in the routing tree: the damage cost is different if a node is a leaf or closer to the root;
- The node position in the dependency graph in our DataIDS (as explained in Section 4);
- The number of nodes under attack (i.e., the cluster in the dependency graph);
- The importance of the data damaged;
- The time and signaling required to perform a countermeasure.

Attack cost is more difficult to evaluate because we must consider some features that are unknown a priori, such as the time needed to perform the attack, the required skills, and the cost to buy a particular equipment. All these elements are strictly dependent on the particular IoT device vulnerability and hardware availability. However, we can assume that the hardware needed for the attack is affordable (sensors are low cost normally), while for the time and skills, we expect high costs because we can assume that the device firmware does not contain simple and easily exploitable flaws. The damage and attacks are summarized in Table 2.

According to the damage and the attack costs, we can accept the risk or take proper countermeasures to mitigate the attack. As an example, we can accept the risk when the attack and countermeasure costs are high but the damage cost is low. In case of a likely attack, we must either apply a countermeasure or increase the attack cost, e.g., by removing the vulnerabilities that lead to that particular attack.

As an example, if in a Fog/IoT network there are several temperature sensors and only one is under attack, we can evaluate if we can accept the risk that the attack propagates and simply apply a low-cost countermeasure by discarding data from the device under attack or by isolating the node and by reauthenticating it. Instead, if the device is a central node which routes data towards the FU, we need, e.g., to apply network reconfiguration with higher time and energy costs [19]. Therefore, the countermeasure must be correlated to the attack according to the assessed risk outcomes.

### 3.2. Attack Models

Let us consider an IoT system composed by *N* IoT units $U=\{u^{1},\ldots ,u^{N}\}$, each endowed with one sensor.

Without loss of generality, we assume that units in *U* are synchronous, i.e., at each time instant *t* is created, a vector of scalar measurements $x_{t}=\left\{x_{t}^{1},\ldots ,x_{t}^{N}\right\}$ with $x_{t}^{i}\in R_{x^{i}}\subset R$, i∈{1,…,N}, and $R_{x^{i}}$ is the range of allowed values from sensor *i*. This assumption can be relaxed by using appropriate data processing techniques (e.g., interpolation, re-synchronization, etc.). We do not make any assumption about the process generating the data stream $x_{t}$, which is considered unknown a priori. We emphasize that we are not assuming the stationary of $x_{t}$ that might evolve following the dynamic of the physical phenomenon monitored.

It is worth stressing that, differently from the literature where the homogeneity or monotonicity assumption is considered [4,12], our work units in *U* might be weakly or strongly related to each other, i.e., sensors can be heterogeneous (they measure different physical quantities, e.g., temperature and humidity).

We only assume that our Fog/IoT system initially behaves in attack-free situations; an attack might occur only later during the system lifetime. This assumption reflects the fact that an attack requires some time to be performed and that we can assume that the system is behaving as intended at the beginning of our modeled period.

We consider the case where a subset $U_{A}$ of units, with $U_{A}\subset U$, could be gained by an attacker, modifying data coming from units in $U_{A}$ as follows:

(1) $$x_{t}^{j}=\left\{\begin{array}{cc} x_{t}^{j} & t<t_{j}^{*}\\ f_{\theta_{j}}\left(x_{t}^{j}\right), & t\geq t_{j}^{*}, \end{array}\right.$$

where $u_{j}\in U_{A}$, $f_{\theta_{j}}\left(x_{t}^{j}\right)$ models the (possibly time-variant) perturbation affecting $u_{j}$, and $t_{j}^{*}$ is the on-set attack time of $u_{j}$.

We model four different types of malicious data injections, i.e., stuck-at, replay, and sensor replacements that are dived in two sub-cases: noise addition and dynamic perturbation attacks to IoT units.

- *Stuck-at:* the attacker gains access to unit $u_{j}$ at time $t_{j}^{*}$ and replaces the values $x_{t}^{j},t\geq t_{j}^{*}$, with the constant value $x_{t_{j}^{*}}^{j}$, i.e.,
  (2) $$f_{\theta_{j}}\left(x_{t}^{j}\right)=x_{t_{j}^{*}}^{j},\;t\geq t_{j}^{*};$$
- *Replay:* the attacker gains access to unit $u_{j}$ at time $t_{j}^{*}$ and replaces the values $x_{t}^{j},t\geq t_{j}^{*}$ with the data acquired up to time $t_{j}^{*}$, i.e.,
  (3) $$f_{\theta_{j}}\left(x_{t}^{j}\right)=\Pi \left(t,t_{j}^{*}\right),\;t\geq t_{j}^{*},$$
  where $\Pi \left(t,t_{j}^{*}\right)$ models the repetition at time *t* of data acquired before time $t_{j}^{*}$;
- *Sensor replacement (noise addition):* the attacker gains access to unit $u_{j}$ at time $t_{j}^{*}$ and introduces a random perturbation to the values $x_{t}^{j},t\geq t_{j}^{*}$, i.e.,
  (4) $$f_{\theta_{j}}\left(x_{t}^{j}\right)=x_{t}^{j}+\eta_{j},\;t\geq t_{j}^{*};$$
  where $\eta_{j}$ is an independent and identically distributed random variable accounting, e.g., for an additional noise affecting the original measurement $x_{t}^{j}$;
- *Sensor replacement (dynamic perturbation):* the attacker gains access to unit $u_{j}$ at time $t_{j}^{*}$ and perturbs the values $x_{t}^{j},t\geq t_{j}^{*}$ by modifying the signal dynamic, i.e.,
  (5) $$f_{\theta_{j}}\left(x_{t}^{j}\right)=\left(1+\delta \right)\;\cdot \;x_{t}^{j},\;t\geq t_{j}^{*};$$
  where δ∈R accounts for the magnitude of the perturbation.

**Figure 3:**
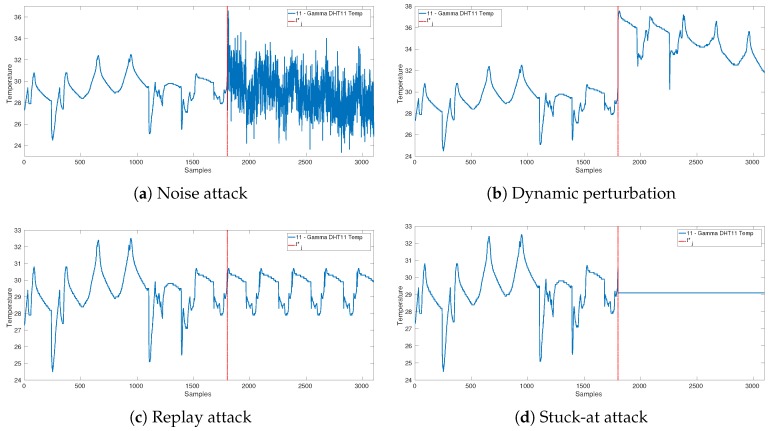
Examples of the attack models at sampling time $t_{j}^{*}=1800$ acquired in our test bed.

Figure 3 shows an example for each of these four types of considered attacks. For instance, those attack types would be realized by an attacker if they substitute or modify the code installed in the sensors with a malicious one.

Our goal is to analyze the datastreams $x_{t}$ to promptly identify and isolate an attack affecting *U*.

## 4. DataIDS

The idea of the proposed attack detection and isolation mechanisms is to characterize the relationships existing among the acquired datastreams and to analyze them over time, looking for an unexpected behavior of one node. In more details, the proposed algorithm relies on an initial data sequence $D_{S}$ storing the first *S* samples acquired by all the sensors $u_{i}\in U$, i.e.,

(6) $$D_{S}=\left[\begin{array}{ccc} x_{1}^{1} & \cdots & x_{S}^{1}\\ \vdots & \ddots & \vdots \\ x_{1}^{N} & \cdots & x_{S}^{N} \end{array}\right].$$

where $D_{S}$ represents the measurements (e.g., temperature or humidity) acquired in an initial attack-free situation. Tt is partitioned into training set $T_{S}$ and validation set $V_{S}$, where $T_{S}$ stores the first *T* samples acquired by all the sensors and $V_{S}$ stores the remaining S–T samples, i.e.,

(7) $$T_{S}=\left[\begin{array}{ccc} x_{1}^{1} & \cdots & x_{T}^{1}\\ \vdots & \ddots & \vdots \\ x_{1}^{N} & \cdots & x_{T}^{N} \end{array}\right],\;V_{S}=\left[\begin{array}{ccc} x_{T+1}^{1} & \cdots & x_{S}^{1}\\ \vdots & \ddots & \vdots \\ x_{T+1}^{N} & \cdots & x_{S}^{N} \end{array}\right].$$

$T_{S}$ is used to learn the relationships among the sensors in *U* by analyzing the cross correlation between the respective data streams $x_{t}$. To achieve this goal, we rely on the concept of *dependency graph* [20] that has been introduced to capture and model the relationships among sensors. A dependency graph is an undirected graph G={N,E}, where nodes N represent the N sensors in *U* and edges E represent the relationships between couples of sensors. In our specific case, the edge $e_{i,j}$ between $u_{i}$ and $u_{j}$ exists in E when

(8) $$r_{i,j}^{T}>\gamma$$

where $r_{i,j}^{T}$ is a *cross-correlation index* measured as the normalized absolute value of the peak of the cross correlation between the data sequences $\left\{x_{1}^{i},\cdots ,x_{T}^{i}\right\}$ and $\left\{x_{1}^{j},\cdots ,x_{T}^{j}\right\}$ and where γ∈[0,1] represents the user-defined threshold value for the cross-correlation index. Such a value has a statistical interpretation representing the minumum value of the cross correlation between two datasequences to create an edge in the dependency graph. γ could range from 0.8 to 0.99 (i.e., from 80% to 99%). In the experimental analysis described in Section 5, γ has been set to 0.9.

The idea of using the cross correlation resides in the ability to define an index, i.e., a scalar index bounded between −1 and 1, characterizing the relationship between the functional behaviors of two data streams. In such a way, we can move the analysis between data streams to the analysis of a scalar value for each couple of data streams.

**Figure 4:**
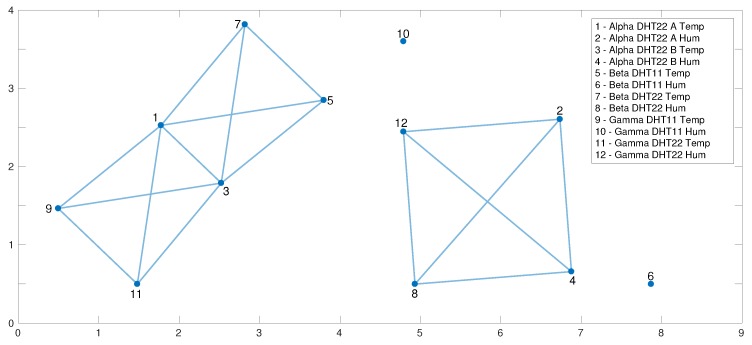
The estimated dependency graph for the considered test bed.

An example of a dependency graph, built from our test bed (described in Section 5.1) is shown in Figure 4. The edges are particularly interesting because they show that temperature sensors (i.e., nodes $u_{1}$, $u_{3}$, $u_{5}$, $u_{7}$, $u_{9}$, and $u_{11}$) are related each other, and the humidity sensors (i.e., nodes $u_{2}$, $u_{4}$, $u_{8}$, and $u_{12}$ related to DHT22 sensors in the test bed) exhibit a similar behavior. Differently, other humidity sensors (i.e., nodes $u_{6}$, $u_{10}$, and DHT11 sensors in the testbed) are not related with any of the sensors. Moreover, we do not detect relationships in the dependency graph between temperature and humidity sensors even if they are close in position. This validates the accuracy of our framework also in terms of heterogeneity among the sensors. We emphasize that the physical position of sensors is not considered in the building of a dependency graph that only comprises the information content present in data, i.e., two physically close sensors are connected through an edge in the dependency graph only if they are cross correlated according to Equation (8). For example, if the attacked sensor is the number 11, it is placed in the same position as number 9 (and with sensors 10 and 12), as shown in Figure 7, but in the dependency graph (in Figure 4), it is also related with 1 and 3, that are in another location. We emphasize that the absence of an edge connecting sensors 6 and 10 does not mean that those two sensors are not related to the other sensors. It means that the cross correlations they have with the other sensors is below the threshold value γ.

This is an example of a dependency graph derived for a test bed with a limited number of sensors; however, the dependency graph shows the relationships of each device with the other ones and does not depend on the number of sensors considered. If there is a high number of sensors, it is possible to expect an increased calculation time to build the dependency graph at the beginning.

### 4.1. Attack Detection

Once the dependency graph has been computed, FU monitors the relationships of each sensor with the “most related” sensors (as shown in the dependency graph) over time, searching for changes. It checks for changes in the cross-correlation index only for the sensors connected with the considered sensor in the dependency graph (i.e., the most “related” in terms of cross-correlations index). Here, changes refer to attacks perturbing the acquired data streams as previously shown.

In more detail, let $C_{i}$ be the set of sensors connected to $u_{i}$ according to the dependency graph. At each time instant t>S, the following *change-detection index* is calculated:

(9) $$R_{i}\left(t\right)=\sum_{j\in C_{i}}r_{i,j}^{t,W},$$

where $r_{i,j}^{t,W}$ is the cross-correlation index defined above and computed over the last *W* recently acquired samples $\left\{x_{t-W+1}^{i},\cdots ,x_{t}^{i}\right\}$ and $\left\{x_{t-W+1}^{j},\cdots ,x_{t}^{j}\right\}$ coming from sensors $u_{i}$ and $u_{j}$, respectively.

A detection occurs when

(10) $$R_{i}\left(t\right)<\Theta_{d}^{i}$$

where $\Theta_{d}^{i}$ is an automatically computed threshold defined as

(11) $$\Theta_{d}^{i}=M^{i}-\lambda_{d}\left(M^{i}-m^{i}\right)$$

and

(12) $$\begin{array}{cc} M^{i} & =\frac{1}{S-T}\sum_{t=T+1}^{S}R_{i}\left(t\right), \end{array}$$

(13) $$\begin{array}{cc} m^{i} & =\min\{R_{i}\left(T+1\right),\ldots ,R_{i}\left(S\right)\}, \end{array}$$

and $\lambda_{d}>1$ is a user-defined parameter representing a confidence parameter for the detection phase acting as a multiplier coefficient in computing the threshold for detecting changes. The larger $\lambda_{d}$, the smaller the threshold, i.e., a smaller threshold would reduce false-positive detections but at the expenses of the (possible) increase of false negative detections and detection delays.

**Figure 5:**
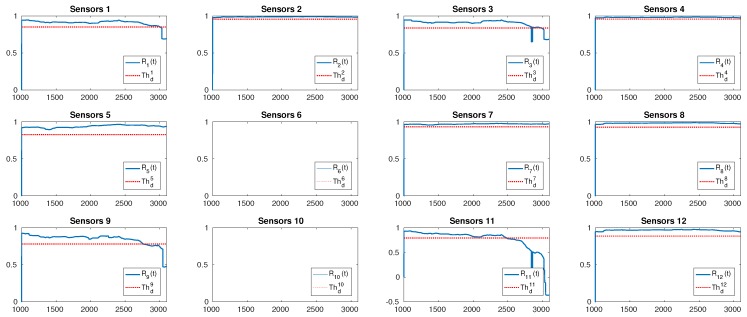
An example of change detection analysis carried out on cross-correlation indices $R_{i}\left(t\right)$ of all the sensors when the attacked sensor is $u_{11}$. The first S=1000 samples belong to the training sequence. In each sub-figure, on the x-axis, we have the samples and, on the y-axis, we have the cross-correlation index. In the legends, the symbol ${\mathrm{Th}}_{d}^{i}$ corresponds to $\Theta_{d}^{i}$ in the text.

An example of detection is given in Figure 5. Here, the attacked sensor is $u_{11}$ at time $t_{j}^{*}=1800$ and, as expected, by looking at the dependency graph in Figure 4, the cross-correlation indices $R_{i}\left(t\right)$ computed in sensors $u_{1}$, $u_{3}$, $u_{9}$, and $u_{11}$ perceived a change. The cross-correlation indices $R_{i}\left(t\right)$sin the other sensors do not exhibit changes.

From the technological point of view, the IoT devices are constrained in memory, computation, and energy and, for this reason, the detection step is carried out by the FU layer, where more computation and storage resources are available. The use of FU leads to less network overhead, but it also increases the attack risks (if the same FU is attacked). However, if the IoT devices have processing capabilities to calculate cross-correlation indices, the change-detection phase can be implemented in a distributed way directly at the IoT devices, under the assumption that the sensors connected in the dependency graph can exchange the acquired information. Hence, each sensor in *U* monitors its own $R_{i}\left(t\right)$ over time and the first sensor detecting a change according to Equation (9) raises an alarm and activates the next isolation phase. We emphasize that, in both cases, the change-detection phase is carried out for sensors for which $C_{i}\neq \emptyset$, i.e., the sensor must be related to at least one of the other sensors in *U* according to the dependency graph. When $C_{i}=\emptyset$, as in the case of sensors $u_{6}$ and $u_{10}$, the analysis based on cross correlation cannot be considered and one could resort on change-detection analysis based on inspection of the residual between the output of a suitably trained prediction model (e.g., linear input–output models or recurrent neural networks) on the acquired data (see, for example, Reference [21]).

### 4.2. Attack Isolation

Once an attack has been detected on an IoT device in *U*, the isolation procedure is activated to identify the device representing the target of the attack. We emphasize that, thanks to the analysis of cross correlations, the sensor with the change in cross-correlation index could not be the one attacked (and, in most cases, the attacked sensor could be interested in not raising an alarm at all).

With $u_{\hat{i}}$ as the sensor with the cross correlation changing at time $\hat{t}$, the isolation procedure analyses acquired data from $\hat{C}=\left\{u_{\hat{i}}⋃C_{\hat{i}}\right\}$, with $C_{\hat{i}}$ as the set of sensors connected to $u_{\hat{i}}$ according to the dependency graph up to time $\hat{t}$ to identify the attacked IoT device. The isolation procedure must run on the FU, being able to store a large number of data and to execute more computationally demanding procedures.

More specifically, the isolation procedure removes one IoT device at a time from $\hat{C}$ and analyses the behavior of the cross correlation among the remaining sensors, i.e.,

(14) $${\hat{R}}_{i}\left(t\right)=\sum_{j,k\neq i\in \hat{C}}r_{j,k}^{t,W},\;t=S+1,\ldots ,\hat{t},$$

for all $i\in \hat{C}$. The cross-correlation isolation index ${\hat{R}}_{i}\left(t\right)$ for $t=S+1,\ldots ,\hat{t}$ is inspected, looking for changes by relying on an automatically computed threshold defined as

(15) $$\Theta_{is}^{i}=M^{i}-\lambda_{is}\left(M^{i}-m^{i}\right)$$

where $1<\lambda_{is}<\lambda_{d}$ is a user-defined parameter. Once we remove sensor $u_{i}$ from $\hat{C}$ and compute ${\hat{R}}_{i}\left(t\right)$, two different situations arise:

- ${\hat{R}}_{i}\left(t\right)<\Theta_{is}^{i}$ for $t=S+1,\ldots ,\hat{t}$: the set of sensors $\hat{C}-u_{i}$ includes the attacked sensor since the cross-correlation isolation index still shows a decreasing behavior (revealing that the sensor providing perturbed behavior is still in $\hat{C}-u_{i}$);
- ${\hat{R}}_{i}\left(t\right)>\Theta_{is}^{i}$ for $t=S+1,\ldots ,\hat{t}$: the sensor $u_{i}$ can be safely considered the target of the attack, since its removal from $\hat{C}$ prevents the decrease of ${\hat{R}}_{i}\left(t\right)$, meaning that the considered data sequences still exhibit the expected behavior.

In addition, once the attacked sensor ${\hat{u}}_{i}$ has been isolated, the isolation procedure also computes an estimate $\hat{t}$ of the time instant $t_{j}^{*}$ when the attack occurred. $\hat{t}$ is computed by averaging the largest time instant for which ${\hat{R}}_{i}\left(t\right)\leq \Theta_{is}^{i}$ for all the sensors $\hat{C}-{\hat{u}}_{i}$.

**Figure 6:**
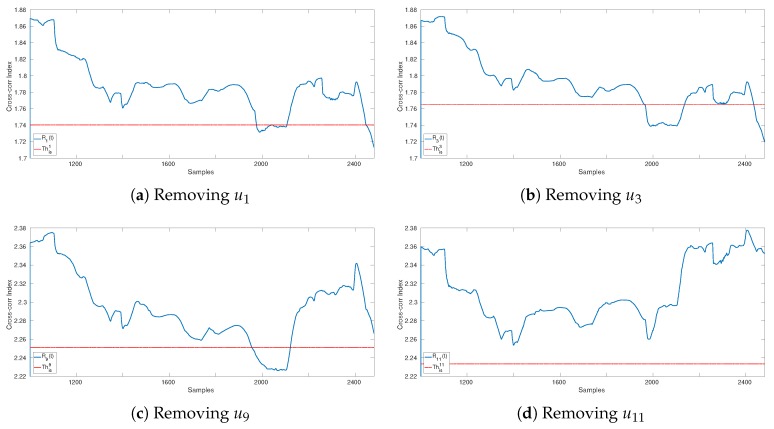
An example of isolation when the attacked sensor is $u_{11}$: The detection has been raised by sensor $u_{9}$ at time $\hat{t}=2481$. The first 1000 samples belong to the training sequence, i.e., S=1000. In the legends, the symbol ${\mathrm{Th}}_{\mathrm{is}}^{i}$ corresponds to $\Theta_{\mathrm{is}}^{i}$ in the text.

An example of isolation is given in Figure 6, where the attacked sensor is $u_{11}$. The detection has been raised by sensor $u_{9}$ at time $\hat{t}=2481$. Here, $\hat{C}=\left\{u_{1},u_{3},u_{9},u_{11}\right\}$ and no detection occurs for ${\hat{R}}_{11}\left(t\right)$, t=1001,…,2481, meaning that $u_{11}$ is the attacked sensor. Conversely, ${\hat{R}}_{1}\left(t\right)$, ${\hat{R}}_{3}\left(t\right)$, and ${\hat{R}}_{9}\left(t\right)$ raise detection before $\hat{t}=2481$.

When none of the IoT devices in $\hat{C}$ was revealed to be the target of the attack, i.e., no detection occurred in any of the sensors in $\hat{C}$, our isolation procedure was not able to isolate the attacked sensor. In this case, a general “attack alarm” message is raised, signaling that one of the sensors in $\hat{C}$ has been attacked.

We emphasize that the proposed isolation procedure implicitly assumes that only one IoT device in $\hat{C}$ has been attacked. If multiple sensors are under attack, this assumption can be weakened by forcing the dependency graph to create clusters of IoT devices characterized by smaller cardinalities to isolate the only sensor compromised.

It is worth noting that this approach is not able to divide attacks from faults, but if we implement the relative countermeasures and the algorithm is triggered very quickly, it is certainly a sensor fault.

## 5. Experimental Results

In this section, we describe the test bed used to validate the methods presented in Section 4.

### 5.1. Description of the Test Bed

The test bed at the University of Florence is made by 6 DHT11–DHT22 devices by Aosong, generating 12 independent humidity and temperature data streams. DHT11 and DHT22 are low-cost environmental devices and are made of two parts: a thermal resistor and a capacitive humidity sensor. Details about the used sensors are summarized in Table 3. Each sensing device is connected to a Raspberry Pi3 which is responsible for data collection.

**Figure 7:**
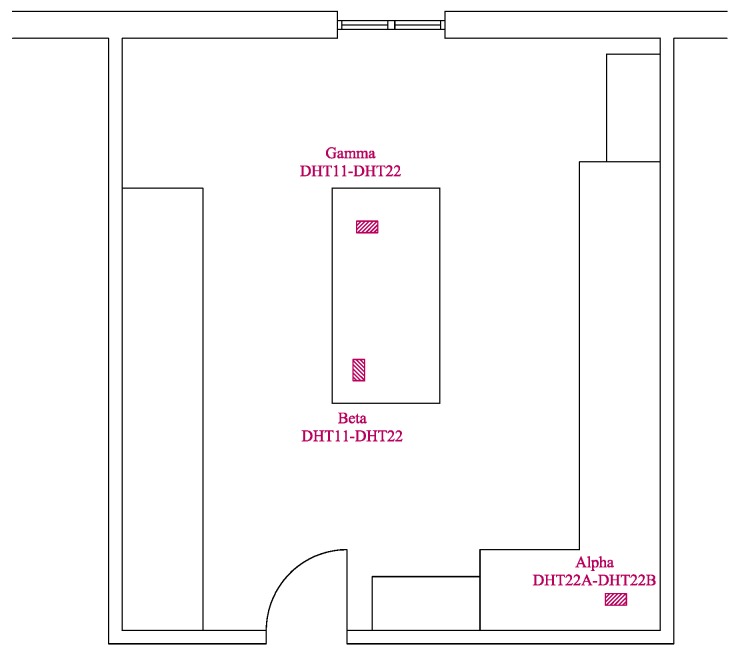
Laboratory floor map and devices positions.

We want to emphasize that our measurements are obtained in a real context. The devices are placed in different positions characterized by an unevenly distributed air conditioning system. As a result, the readings are expected to be similar but not identical. The assignment (i.e., name sensor and type of sensors) is summarized in Table 4, and the map of the Raspberry Pi3 positions is shown in Figure 7.

**Figure 8:**
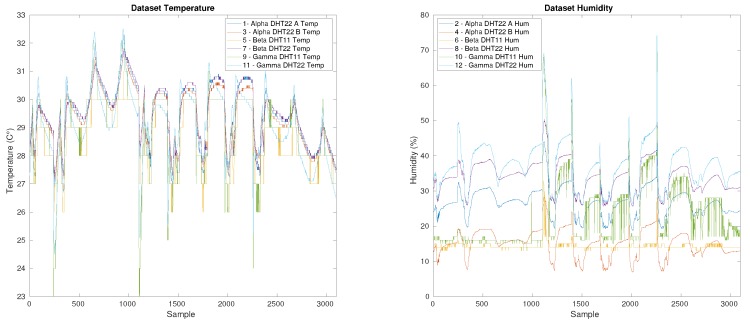
Measured temperature and humidity dataset.

The configuration of the system and the dataset (about 10,430 samples) acquired every 5 minutes are available at the link https://www.gaucho.unifi.it. The dataset is shown in Figure 8.

### 5.2. Description of the Considered Attacks

We defined four different attacks according to the models shown in Section 3.2:

- *Stuck-at*, where $x_{t}^{j}=x_{1800}^{j},t\geq t_{j}^{*}$;
- *Replay*, where $\Pi \left(t,1800\right)$ replaces $x_{t}^{j},t\geq t_{j}^{*}$ with values acquired 24 hours before $t_{j}^{*}=1800$;
- *Sensor replacement (noise addition)*, where $\eta_{j}=N\left(0,1.5\right)$ is a Gaussian random variable with zero mean and standard deviation equal to 1.5;
- *Sensor replacement (dynamic perturbation)*, where the magnitude of the perturbation is δ=0.2.

Every attack starts at t=1800. Each attack is repeated on all sensors (one at a time) for a total of 48 experiments.

### 5.3. Figures of Merit

In order to evaluate the effectiveness of the proposed algorithm, we defined the following seven figures of merits:

- *Attack detected*: binary value describing whether the attack has been detected (1) or not (0);
- *Detection counter*: number of sensors within the network that detected an attack (excluding the attacked sensor);
- *Min detection time*: when the first sensor detected an attack within the network;
- *MW detection time*: when at least half the sensors connected to the sensor under the attack detect the attack;
- *Max detection time*: when the last sensor detected an attack within the network;
- *Isolated attack*: binary value describing whether the attack has been correctly isolated (1) or not (0);
- $\hat{t}$: estimation time.

### 5.4. Experimental Results

The experimental results are summarized in Table 5. The four types of cyber attacks described in Section 5.2 have been applied to all the IoT devices in the test bed, i.e., #1–#12. In this experimental analysis the parameters of the proposed solution have been set as in Table 6.

Cyber attacks have been detected in 97.5% of the cases, i.e., 39 of the “detected attacks” over 40 experiments. We emphasize that we did not experience false-positive detection, equivalent to a precision of 100% and to a recall of 97.5%. The isolation capability worked in 82.5% of the cases, i.e., 33 of the “isolated attacks” over 40 tests, which is still a very good result. We emphasize that, in those cases where a correct isolation is not achieved, the proposed solution is not able to isolate an attacked sensor. Hence, in the considered experimental analysis, the proposed solution is either able to correctly isolate the attacked sensor or it does not provide any isolation (i.e., we do not isolate wrong sensors). This lead to a precision of 100% and to a recall of 82.5%

For what concerns the sensibility of the proposed methods to the attack type, it is worth observing that the *replay*, *stuck-at*, and *dynamic perturbation* attacks have been correctly detected and isolated with extreme high accuracy (100% detection and 96.7% correct isolation). The most difficult attack to deal with is the *noise addition* attack, where the detection still performs well (90% of detection) but the isolation presents poor performances (40% correct isolation).

The results about the “detection counter” show the efficiency of the proposed distributed analysis. In almost all *replay*, *stuck-at*, and *dynamic perturbation* attacks, all the IoT devices belonging to the cluster where the specific sensor has been attacked detect the change. Even in the case of a *noise addition* attack (the most critical for the isolation procedure), the “detection counter” is larger than or equal to 1 even when the attacked IoT device cannot been isolated successfully. This means that the proposed algorithm is able to detect the presence of the attack at the cluster level even when it was not able to specifically isolate the attacked IoT device.

The detection times (i.e., min, MW, and max detection times) show the ability of the proposed solution to promptly detect the presence of an attack. The results about $\hat{t}$, i.e., the estimate of the time instant when the attack started, show excellent capability of the proposed solution to correctly estimate when a given attack started in a given sensor. This allows to discard the erroneous values transmitted by the attacked sensor, obtaining a cleaner dataset.

We also evaluated the average computational time for the creation of the dependency graph and the detection/isolation phases of the proposed solution. The reference hardware platform is a 2.5-GHz Intel Core i7 with 16-GB RAM at 2133-MHz LPDDR3. Creating the dependency graph is the most time-consuming process and requires (on average) 36.2 s, while the average computational time per unit of the detection and isolation phases is 26.4 ms. We want to stress that the creation of the dependency graph can be done in the FU and that the detection and isolation phases can be performed even by a small device like a Raspberry Pi.

### 5.5. Countermeasures

In order to evaluate the countermeasure-decision process, we will assume that an IoT device in the network is being attacked (e.g., sensor #11, Gamma DHT22-Temp). The attack has been successfully detected, and we must decide the appropriate countermeasure to be applied. As shown in Figure 2, we divided the attacks into families depending on the attack type: stuck-at and replay are a (probable) consequence of an attack to the sensor software, while noise addition and dynamic perturbation are most probably related to sensor replacement. Without loss of generality, all the value costs will be in the range [0–10], where 10 is the maximum value.

The attack cost is summarized in Table 7, and it assumes that the attacker is able to physically access the nodes. Moreover, we assume that replacing a node is more difficult than tampering with an existing one. As a matter of fact, using software vulnerability should be easier than gaining access to the network and replacing an existing device (without triggering an immediate alarm).

The damage costs for the related IoT device are shown in Table 8. Here, we do not consider the data relevance, since in our system, all collected data are equally significant.

The possible countermeasures costs are summarized in Table 9. Note that a higher security countermeasure should imply also the use of the lower level ones.

The adopted countermeasure will have total cost less or equal to the damage cost: in our example, when an attack is detected, we can change the MAC-16 (MAC short) [19,22] address of our devices and, if a new attack is perform, we can modify the routing algorithm tree with a complete network reconfiguration.

From the analysis, it is possible to conclude that an address refresh (e.g., by using the techniques outlined in References [19,22]) is a valid countermeasure. On the contrary, if the sensor is more central in the dependency graph (i.e., sensors #1 and #3), the appropriate countermeasure would be to apply an address refresh and a routing reconfiguration. Cryptography key renewal will be used as a last resort in case of an attack to sensors generating important data or if the attack persists after the network reconfiguration.

## 6. Concluding Remarks

In this paper, we proposed a novel IDS based on the analysis of data acquired in real-time by different Fog/IoT devices. DataIDS can promptly detect a cyber attack affecting a device of the FC/IoT system as well as effectively isolate it within the network to support the reaction phase. In order to react to the attack, we propose an attack-tree-based evaluation system, which has the advantage of avoiding countermeasures that are disproportionate with respect to the attack and the damage costs.

We like to stress that the proposed system can be used also to strengthen the robustness of a Fog/IoT system against attacks.From the dependency graph, it is in fact possible to highlight 1) the nodes that are unconnected and 2) the nodes with high correlation indices. In the first case, the nodes are either collecting outlier measures (thus, discardable) or important measures (thus, more nodes should be installed in that particular point). It is obvious that nodes with high correlation indices, i.e., more related in the dependency graph, should be more protected. As a consequence, it is possible to choose the best candidate nodes to be, for example, hardened by physical security measures (e.g., anti-tampering hardware).

We implemented a test bed to validate the performance of DataIDS on a real dataset and, as shown by our results, the proposed intrusion detection system has several advantages over other kinds of approaches, and it can be easily implemented in constrained resource devices.

In future works, we plan to extend our model in order to better address the problem of data privacy by using fog devices. This will allow to maintain user data privacy while enabling cooperative intrusion detection capabilities among different and logically separated sensors zones [23]. Moreover, we plan to study how the attack detection and isolation capabilities are influenced by the dependency graph properties (e.g., size, number of connected vertex, etc.).

## Figures and Tables

**Table 1:** Attack detection comparison.

	Traditional IDS	DataIDS
Jamming & Replacement	Difficult	Yes
Modify Environment	No	Yes
Replace Component	No	Yes
Physical Tampering	No	Yes
Bribe Network Administrator	No	Yes (if data are modified)
Exploit Network Threat	Yes	Yes (if data are modified)

**Table 2:** Damage and attack costs.

	Routing tree position
**Damage**	Number of nodes (cluster) under attack
	Dependency graph position
	Data importance
	
**Attack**	Cost to find the attack
	Time required for the attack
	Equipment cost
	Skill required
	Physical access to the nodes
	Attack reproducibility

**Table 3:** DHT 11–DHT 22 details.

	Max Sampling Rate	Type	Readings Interval	Accuracy
DHT 11	1 Hz	Humidity	(20 ÷ 80) %	5 %
		Temperature	(0 ÷ 50) °C	±2 °C
DHT 22	0.5 Hz	Humidity	(0 ÷ 100) %	(2 ÷ 5) %
		Temperature	(−40 ÷ 80) °C	±0.5 °C

**Table 4:** Test bed sensors names and types.

Pi3	Sensors	Number	Type
Alpha	DHT22 A	#1	Temperature
		#2	Humidity
	DHT22 B	#3	Temperature
		#4	Humidity
Beta	DHT11	#5	Temperature
		#6	Humidity
	DHT22	#7	Temperature
		#8	Humidity
Gamma	DHT11	#9	Temperature
		#10	Humidity
	DHT22	#11	Temperature
		#12	Humidity

**Table 5:** Experimental results on the considered dataset: The symbol ✓ means that the attack has been detected/isolated, while the symbol – means that the attack has not been detected/isolated.

	Sensor	#1	#2	#3	#4	#5	#7	#8	#9	#11	#12
Replay Attack	Detected Attack	✓	✓	✓	✓	✓	✓	✓	✓	✓	✓
	Detection counter	5 / 5	3 / 3	5 / 5	3 / 3	3 / 3	3 / 3	3 / 3	3 / 3	3 / 3	3 / 3
	Min Detection Time	2088	2144	2091	2160	2542	2862	2151	2442	2760	2150
	MW Detection Time	2783	2256	2824	2254	2747	3022	2168	3095	2840	2166
	Max Detection Time	3086	2440	3072	2472	2867	3043	2454	3095	3006	2260
	Isolated Attack	✓	✓	✓	✓	–	✓	✓	✓	✓	✓
	$\hat{t}$	2009.9	2063.3	2027.4	2055.3	–	2253.6	2068.3	2162.1	2291.4	2114.0
Stuck-At Attack	Detected Attack	✓	✓	✓	✓	✓	✓	✓	✓	✓	✓
	Detection Counter	5 / 5	3 / 3	5 / 5	3 / 3	3 / 3	3 / 3	3 / 3	3 / 3	3 / 3	3 / 3
	Min Detection Time	1865	1911	1863	1925	1857	1950	1900	1924	2431	1938
	MW Detection Time	1952	2114	1945	1980	1938	1955	1939	2181	2694	1974
	Max Detection Time	1964	2137	1961	2133	1954	1967	2136	2241	2718	2122
	Isolated Attack	✓	✓	✓	✓	✓	✓	✓	✓	✓	✓
	$\hat{t}$	1816.0	1854.0	1818.0	1839.7	1831.1	1830.2	1856.7	1843.7	1909.4	1886.3
Noise Addition Attack	Detected Attack	✓	✓	✓	✓	✓	✓	✓	✓	✓	–
	Detection Counter	5 / 5	1 / 3	5 / 5	1 / 3	2 / 3	2 / 3	1 / 3	1 / 3	2 / 3	0 / 3
	Min Detection Time	1974	2795	1920	3055	1973	2715	3067	2062	2431	–
	MW Detection Time	2473	–	2431	–	2749	2747	–	–	2734	–
	Max Detection Time	2742	2795	2493	3055	2749	2747	3067	2062	2734	–
	Isolated Attack	✓	–	✓	–	–	✓	–	–	✓	–
	$\hat{t}$	1846.6	–	1834.4	–	–	1847.0	–	–	1863.6	–
Dynamic Perturbation Attack	Detected Attack	✓	✓	✓	✓	✓	✓	✓	✓	✓	✓
	Detection Counter	5 / 5	3 / 3	5 / 5	2 / 3	3 / 3	3 / 3	3 / 3	3 / 3	3 / 3	3 / 3
	Min Detection Time	1810	1947	1807	2557	1813	1859	1896	1933	1862	1975
	MW Detection Time	1936	2147	1872	2750	1920	1938	1944	2001	1974	2094
	Max Detection Time	2003	2702	1896	2750	1975	1982	2509	2060	2017	2311
	Isolated Attack	✓	✓	✓	✓	✓	✓	✓	✓	✓	✓
	$\hat{t}$	1803.5	1858.3	1802.7	1974.0	1807.0	1804.3	1847.0	1809.2	1803.8	1910.3

**Table 6:** Parameters of the proposed solution and attack models.

$t_{j}^{*}=1800$	Time instant the attack started (for the four models of attacks)
δ=0.2	Magnitude of the perturbation in the sensor replacement (dynamic perturbation) model
γ=0.9	Threshold value for the cross-correlation index in Equation (10)
σ=1.5	Standard deviation of the Gaussian random variable in the sensor replacement (noise addition) model
λ=4	Confidence parameter for detection phase
$\lambda_{is}=1.4$	Confidence parameter for isolation phase in Equation (15)
γ=0.9	Threshold value for the cross-correlation index in Equation (10)

**Table 7:** Attack table cost.

	Stuck-At	Replay	Noise dd.	Dyn. Pert.
Time required	2	5	7	7
Equipment cost	3	3	3	3
Skill required	5	5	7	7
Physical access	4	4	4	4
Average	3.5	4.25	5.25	5.25

**Table 8:** Damage cost for sensor 11.

	Routing	Cluster	Dependency	
	Tree Position	Under Attack	Graph Position	Average
Cost	4	3	2	3

**Table 9:** Countermeasure cost for sensor 11.

	Refresh	Refresh	Change
	Address	Routing Tree	Keys
Signaling Cost	3	5	10
Time Cost	4	6	7
Average	3.5	5.5	8.5
